# Quantitative Microbial Risk Assessment of *Helicobacter pylori* and Enteric Pathogens in Fresh Vegetables in the Central Highlands of Peru

**DOI:** 10.3390/foods15091596

**Published:** 2026-05-05

**Authors:** María Custodio, Richard Peñaloza, Jonathan Crispin-Ayala, Rosa Paredes-Alhua, Ciro Rodríguez

**Affiliations:** 1 Facultad de Medicina Humana, Universidad Nacional del Centro del Perú, Av. Mariscal Castilla N° 3909, Huancayo 12000, Peru; e_2022100747e@uncp.edu.pe (J.C.-A.); crodriguez@uncp.edu.pe (C.R.); 2 Water Research Laboratory, Universidad Nacional del Centro del Perú, Av. Mariscal Castilla N° 3909, Huancayo 12000, Peru; rparedes@uncp.edu.pe; 3 Facultad de Zootecnia, Universidad Nacional del Centro del Perú, Av. Mariscal Castilla N° 3909, Huancayo 12000, Peru; rpenhaloza@uncp.edu.pe

**Keywords:** *Helicobacter pylori*, *Campylobacter jejuni*, enteric bacteria, QMRA, qPCR, vegetables

## Abstract

The rise in global consumption of fresh vegetables is a response to their nutrient-dense composition and low caloric content—key factors for optimising human metabolic health. This study evaluated the Quantitative Microbial Risk Assessment (QMRA) of *Helicobacter pylori* and enteric pathogens in fresh vegetables within the central highlands of Peru. The research integrated conventional microbiology, qPCR, and Monte Carlo simulations. The results revealed a high prevalence of *Escherichia coli* (83.7%), with a heterogeneous distribution where Huancayo presented the highest prevalence (95.5%) and Chupaca the lowest (68.2%). In contrast, pathogens such as *H. pylori* and *Campylobacter jejuni* showed marginal prevalences of 2.33% and 3.49%, respectively, with detections restricted to leafy and root vegetables at specific points of sale. Although biochemical tests indicated the presumptive presence of *Helicobacter pylori*, the qPCR results were negative, possibly due to the bacteria’s viable but non-culturable (VBNC) state. The QMRA model showed a highly skewed annual infection risk distribution, with *E. coli* presenting the highest risk: median Pann = 1.000 and 84.3% of simulations exceeding the WHO tolerable threshold of 10^−4^. For *Salmonella* Typhimurium and *Shigella* flexneri, 22.4% and 9.1% of simulations exceeded the same threshold, respectively. The results underscore the urgent need to implement traceability programs and improve agricultural practices across the evaluated provinces.

## 1. Introduction

The demand for fresh vegetables has grown significantly on a global scale due to their nutritional profile, which provides a high concentration of micronutrients and a low energy density essential for metabolic health [1]. The World Health Organization (WHO) suggests including at least 400 g of fruits and vegetables per day as part of a healthy diet to improve overall health and reduce the risk of non-communicable diseases [2]. Despite these benefits, the fact that they are mostly consumed raw positions these foods as primary transmission routes for enteric diseases [3]. Contamination can stem from deficient agricultural practices and failures in post-harvest disinfection protocols. These pathogens do not merely adhere to the surface; they are capable of internalizing within plant tissues, compromising the efficacy of washing and increasing the risk of gastrointestinal outbreaks [4].

In developing countries, irrigation with wastewater is a common strategy in the face of water scarcity. Nonetheless, deficiencies in post-harvest hygiene facilitate the persistence of gastric [5] and enteric pathogens [6,7,8]. *Helicobacter pylori* is a critical pathogen whose global prevalence reached 43.2% between 2011 and 2022 [9], driven by its persistence in water sources and the food chain. Its relevance is alarming, given that gastric cancer constitutes the fourth leading cause of neoplastic mortality worldwide [10,11]. While person-to-person transmission is the most widely recognized route, recent evidence positions irrigation water and vegetable matrices as critical and underestimated environmental reservoirs. Consequently, the survival of the pathogen or its DNA in these vehicles directly links the consumption of raw vegetables to the prevalence of chronic gastritis and the risk of gastric adenocarcinoma [12]. In the Peruvian context, the microbiological safety of short-stem vegetables constitutes a critical and persistent challenge, stemming primarily from the widespread use of contaminated water sources for agricultural irrigation [13].

Vegetable safety in the Peruvian context is further complicated by region-specific agricultural and climatic factors. The Junín highlands are characterized by smallholder farming systems that rely heavily on glacial meltwater and untreated river water for irrigation, limited access to synthetic fertilizers (necessitating the use of animal manure), and cold, semi-arid inter-Andean valley conditions that may differentially affect pathogen survival. These conditions contrast markedly with the lower-altitude coastal agricultural zones of Peru, where drip irrigation and regulated water sources are more prevalent [13,14]. Understanding these distinctions is critical for designing targeted food safety interventions appropriate to the Andean context.

Internationally, several frameworks have been established to ensure the microbiological safety of fresh produce. In the European Union, Commission Regulation (EC) No 852/2004 mandates the implementation of Good Manufacturing Practices (GMP) and Good Hygiene Practices (GHP) throughout the food chain, with specific microbiological criteria for ready-to-eat vegetables established under Regulation (EC) No 2073/2005 (e.g., *Salmonella* absent in 25 g; *E. coli* ≤ 100 CFU/g for pre-cut vegetables). Hazard Analysis and Critical Control Points (HACCP) systems have been shown to reduce microbial contamination in fresh produce by 1–3 log CFU/g when applied from the field to retail [15,16]. These models represent positive benchmarks that could be adapted for implementation in the Peruvian central region.

Quantitative microbial risk assessment (QMRA) constitutes the standard methodological framework for quantifying the probability of infection and its public health impact. Its systematic approach allows for the integration of variability in exposure data, enabling risk managers to make decisions grounded in scientific evidence [17]. However, although qPCR has streamLined microbiological monitoring, its integration into quantitative risk models requires strict standardization of its analytical limits. Without rigorous control of these technical variables, the uncertainty inherent in molecular detection is transferred to the risk metric, distorting the evaluated epidemiological reality [18]. This problem is exacerbated by critical limitations, such as the presence of inhibitors in plant tissues and the inability to distinguish between viable cells, dead cells, or those in a viable but non-culturable (VBNC) state—factors that introduce significant biases into the quantification of the infectious dose.

In this context, the present study aimed to evaluate the quantitative microbial risk assessment (QMRA) of *H. pylori* and enteric pathogens in fresh vegetables marketed in the central highlands of Peru. To achieve this, a dual approach was employed, integrating microbiological characterization through biochemical and molecular (qPCR) methods with the development of a stochastic risk model. The novelty of this research lies in its modeling of how technical sensitivity influences the estimation of annual infection probability. By doing so, it provides a robust and necessary scientific basis for optimizing surveillance protocols and strengthening food safety regulatory frameworks within Peruvian agriculture.

## 2. Materials and Methods

### 2.1. Description of the Study Area

**Figure 1:**
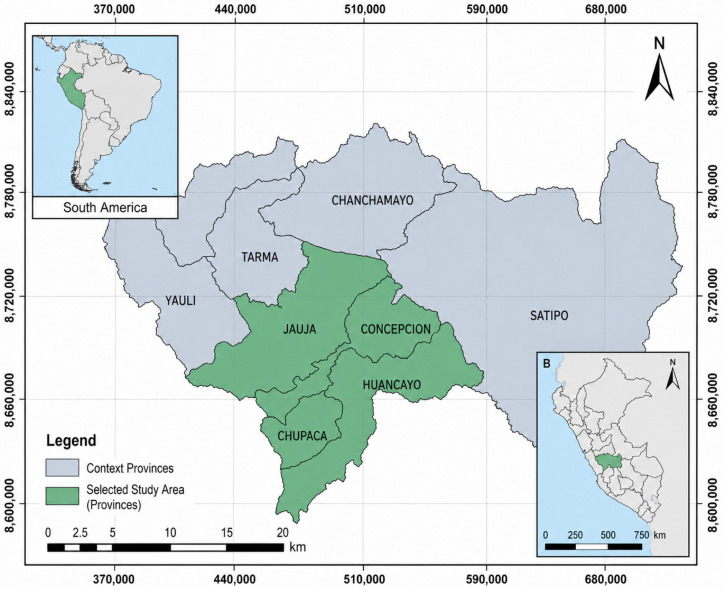
Location map of the study area showing the four selected provinces in the Junín region, Peru.

The study area is in the Junín region at 11°09′32″ S and 75°59′34″ W, specifically within the provinces of Huancayo, Chupaca, Jauja, and Concepción (Figure 1). This region accounts for 3% of the Peruvian territory, covering a surface area of 44,197.23 km^2^. It features a highly rugged terrain due to the Central and Western mountain ranges, with altitudes ranging from 360 to 5000 masl. (meters above sea level). The four selected provinces were chosen based on their agricultural relevance, population density, and representativeness of the central Andean valley system. Huancayo is the regional capital and the most densely populated province, with the highest volume of fresh produce traded in traditional markets. Chupaca, Jauja, and Concepción are predominantly agricultural provinces with distinct microclimatic profiles. Huancayo and Chupaca lie at approximately 3259 masl and are characterized by temperate valley climates (mean annual temperature: 12 °C; annual precipitation: 650–750 mm), while Jauja (3410 masl) and Concepción (3251 masl) share similar climatic profiles but differ in market size and irrigation infrastructure. The provinces were selected over others in the Junín region because the study specifically targets the inter-Andean valley agricultural system, which relies on glacial and river-fed irrigation, in contrast to the warmer, more humid jungle provinces where different contamination dynamics apply.

The climate is variable due to its diverse geography and drastically shifting altitudes. In the inter-Andean valleys, the climate is temperate and cold, with mean annual temperatures ranging between 5 °C and 23 °C. As one descends toward the high jungle and the rainforest in provinces such as Chanchamayo and Satipo, the climate becomes warmer and more humid, with temperatures ranging from 20 °C to 32 °C. Conversely, in the Puna and high-peak zones above 3600 masl, the climate is cold and dry; temperatures vary from 15 °C to −3 °C, with nocturnal frosts that can drop to −20 °C during the winter [19]. Regarding precipitation, Junín has a marked rainy season from November to March or April, which sees the highest rainfall volumes [20]. The dry season extends from May to October, with significantly lower rainfall. Annual precipitation volumes vary considerably by zone: in the inter-Andean valleys of the central highlands, rainfall ranges between 480 mm and 820 mm, while in the high jungle, it is much more abundant, averaging 2600 mm to 4000 mm, and even exceeding 8000 mm per year in some areas.

### 2.2. Vegetable Sample Collection

**Figure 2:**
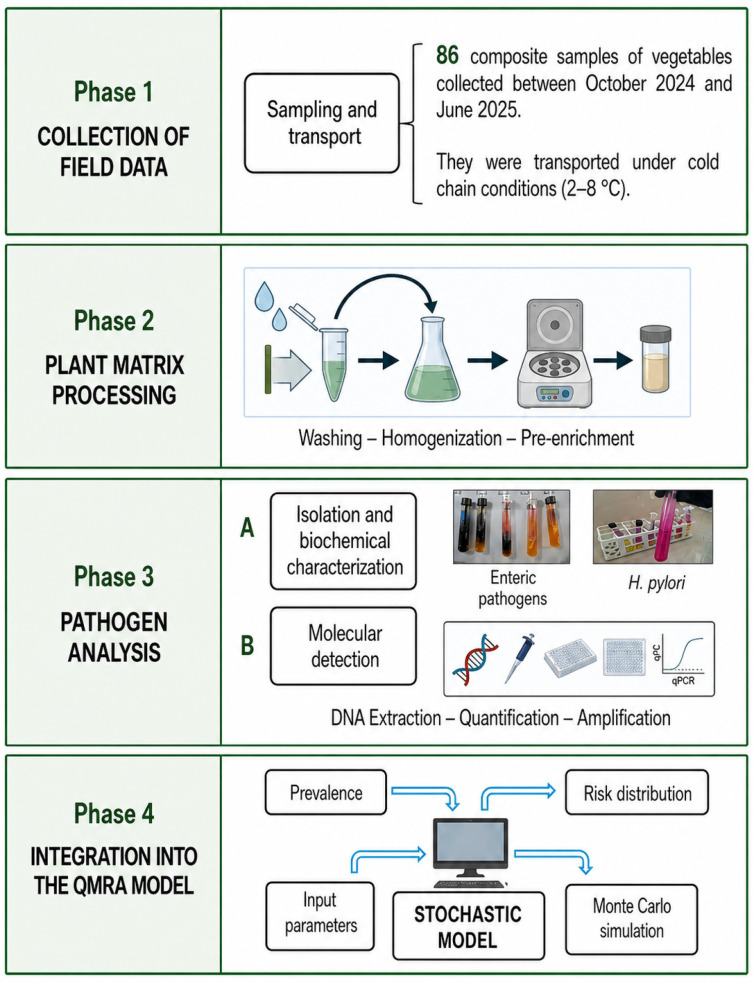
Schematic diagram of the study. Created with BioRender.com.

The study design and methodological workflow are summarized in Figure 2. Between October 2024 and June 2025, a total of 86 composite vegetable samples were analysed: Jauja (n = 22), Concepción (n = 20), Chupaca (n = 22), and Huancayo (n = 22), using a stratified sampling design to ensure the representativeness of the central region of Peru. For each vegetable typology, triplicate 500 g composite samples were obtained through random samples at three distinct points of sale within each market centre. Each composite sample consisted of pooled subsamples from five individual vegetable units of the same species, which were homogenized prior to analysis. This approach was adopted to minimize sampling bias arising from heterogeneous surface contamination. A wide variety of products intended for raw consumption were collected, classified as leafy vegetables, inflorescences, roots and bulbs, and fruits or stems (Table S1). Following collection, the analytical units were divided for specialized processing, where one aliquot was transferred to the Water Research Laboratory for bacteriological analysis and the second was sent to the Molecular Biology Research Laboratory for molecular detection, with all samples strictly transported under cold chain conditions (2–8 °C) to guarantee microbial viability and nucleic acid stability. Upon arrival at the laboratory, samples designated for bacteriological analysis were processed within 2 h of collection. Samples for molecular analysis were stored at −20 °C until DNA extraction, which was performed within 48 h of collection. No significant degradation of nucleic acids was expected under these conditions based on established protocols for frozen vegetable matrices [21,22]. Storage conditions were maintained consistently across all sampling events to minimize any potential effect on microbial viability.

Points of sale were selected within each market using a systematic random sampling approach. Each market was divided into quadrants, and one stall per quadrant was randomly selected per sampling visit. Stalls were identified by the market administration as permanent vendors of fresh produce. This selection strategy ensured geographic coverage within each market and avoided overrepresentation of any single vendor or produce type.

### 2.3. Sample Preparation

From each composite vegetable sample, 25 g were transferred to a homogenization bag containing 225 mL of sterile 0.85% physiological saline solution and subsequently homogenized for 5 min. In the case of whole vegetables—specifically cucumber, carrot, beet, turnip, and radish—each was placed in a bag with the respective volume of sterile physiological saline solution, and the peel was washed by manual rubbing.

The washing procedure followed the protocol described by Denis et al. [23], which specifies manual rubbing for 60 s with 200 mL of sterile physiological saline (0.85% NaCl) per sample. This method is consistent with the approach validated by Ssemanda et al. [24] for the recovery of surface-associated bacteria from fresh produce. No standardized ISO method specifically addresses vegetable surface washing for microbiological analysis; however, the described procedure aligns with the recommendations of ISO/TS 17919:2013 [25] for the detection of foodborne pathogens on fresh produce.

For bacteriological analysis, the homogenized samples were centrifuged at 3000 rpm for 5 min; the resulting sediment was resuspended in 1 mL of Brain Heart Infusion (BHI) broth, diluted with an additional 9 mL of BHI, and incubated at 37 °C for 24 h. The centrifugation speed of 3000 rpm (approximately 900× *g*) was selected to pellet bacterial cells while minimizing co-sedimentation of plant debris. This speed is consistent with the protocol for vegetable matrices and has been validated for the recovery of *H. pylori* from environmental samples [5,26]. Although higher centrifugation speeds can improve cell recovery, they also increase co-pelleting of inhibitory plant compounds. The selected speed represents a validated compromise between bacterial recovery efficiency and matrix purity for downstream culture and molecular analyses. The remaining samples, intended for the molecular analysis of *H. pylori*, were stored at −20 °C until further processing.

### 2.4. Bacterial Isolation, Identification, and Quantification via Culture Techniques

Pre-enriched samples were analyzed to isolate and identify pathogenic bacteria, including *E. coli*, *Salmonella* spp., *Shigella*, *H. pylori*, and *Campylobacter jejuni*. All analyses followed food safety and quality reference guidelines according to ISO methods: ISO 21528-2:2017 for Enterobacteriaceae [27], ISO 16649-2:1999 for *E. coli* [28], and ISO 6579-1:2017/A1:2020 for *Salmonella* spp. [29] and *Shigella* [30]. The isolation of *E. coli* was performed in EC Broth, incubated at 37 °C for 24 h for selective enrichment. Subsequently, a 1 mL aliquot was extracted from each enrichment culture to perform decimal serial dilutions (up to 10^−3^). The inoculum was plated using the surface spread plate technique on MacConkey agar plates, which were incubated at 37 °C for 24 h. The enumeration of *E. coli* was determined by counting typical colonies, expressing final results in colony-forming units per gram of vegetable (CFU/g). A lower microbiological limit of 100 CFU/g corresponds to the maximum acceptable concentration of *E. coli* in fresh vegetables, as established by the Peruvian Ministry of Health [14]. Samples in which bacterial pathogens were detected and confirmed in 25 g of vegetables (or in the whole vegetable), or in which *E. coli* levels were found to be >100 CFU/g, were reported as unacceptable (positive), representing a health risk. The isolation of *Salmonella* and *Shigella* was performed on *Salmonella-Shigella* (SS) agar, and quantification was conducted on Deoxycholate Citrate Agar using decimal serial dilutions (up to 10^−3^). Inoculated plates were incubated at 37 °C for 24 h; typical *Salmonella* colonies appeared colorless with a black center, while *Shigella* colonies did not present a black center. For confirmation of both bacteria, *Salmonella* and *Shigella* colonies were streaked onto Kligler iron agar and incubated at 37 °C for 24 h. Reference strains of E. coli (ATCC 25922) and *Salmonella enterica* subsp. *enterica* serovar Typhimurium GFP (ATCC 14028) (*Salmonella* Typhimurium) was used. Other Enterobacteriaceae identified throughout the isolation process were confirmed via biochemical identification on Kligler iron agar.

The isolation of *Campylobacter* spp. followed the modified International Organization for Standardization (ISO) 10272–2:2017 standards [31]. Homogenized samples were diluted in Bolton broth (10^−1^ dilution) and incubated for 5 h at 37 °C and 44 h at 41.5 °C. Subsequently, serial dilutions were spread on *Campylobacter* agar plates with 8% defibrinated sheep blood and selective supplement (trimethoprim 30 mg/L, polymyxin B 10 mg/L, and vancomycin 10 mg/L). The plates were incubated at 41.5 °C for 44 h under microaerophilic conditions (N_2_: 85%, CO_2_: 10%, and O_2_: 5%). Afterward, a characteristic *Campylobacter* colony was selected, streaked onto sheep blood agar, and incubated under microaerophilic conditions at 37 °C for 36 h ± 2 h. Identification of *Campylobacter* spp. strains was performed using Gram staining and biochemical characterization tests (catalase, oxidase, hippurate hydrolysis, and nitrate reductase production). Reference strains of *Campylobacter* (*Campylobacter jejuni* ATCC 29428, *Campylobacter coli* ATCC 33559) were used [32].

The isolation of *H. pylori* from homogenized samples was carried out following the protocol established by Custodio et al. [33]. Samples were centrifuged at 3000 rpm for 5 min; the supernatant was decanted, and the sediment was resuspended in 2 mL of BHI-Dent for 1 h. Cultivation was performed on modified Brain Heart Infusion blood agar plates, which were selectively supplemented (trimethoprim 30 mg/L and vancomycin 10 mg/L). The plates were incubated at 37 °C under microaerophilic conditions for seven days. At the end of the incubation, the plates were examined to determine the characteristic appearance of *H. pylori* colonies (0.5–1 mm in diameter, circular, convex, translucent, and weakly hemolytic). Biochemical analysis was performed using urease, oxidase, and catalase tests. The *H. pylori* TV1 reference strain was kindly provided by Dr. Vilma Reyes from the Food Microbiology Laboratory of the National University of Central Peru to be used as a positive control in molecular and biochemical assays.

### 2.5. Molecular Detection of Helicobacter pylori

Total genomic DNA extraction was performed from the homogenized samples, beginning with a concentration stage via centrifugation DLAB D3024R (DLAB Scientific Co., Beijing, China) at 6000 rpm for 10 min [21]. Subsequently, a 1000 μL aliquot of each sediment was subjected to a thermal lysis protocol in a dry bath, Thermo Scientific™ (Thermo Fisher Scientific, Waltham, MA, USA) at 98 °C for 4 min, followed by immediate cooling on ice (5 min) to induce thermal shock and cell rupture. The lysates were centrifuged at 8000 rpm for 5 min to separate unlysed cellular material [34]. From 200 μL of the resulting supernatant, DNA was purified using the QIAamp DNA Mini Kit (Qiagen, Germany), strictly following the manufacturer’s specifications. The integrity and concentration of the extracts were determined via microvolume spectrophotometry NanoDrop ONE (Thermo Fisher Scientific, Waltham, MA, USA), ensuring an optimal A260/A280 absorbance ratio for subsequent amplification assays [22].

The molecular detection of *H. pylori* was carried out by amplifying a fragment of the vacA gene (372 bp) using quantitative real-time polymerase chain reaction (qPCR). H. pylori-specific vacA primers were used: Vac1 (5′-GGCACACTGGATTTGTGGCA-3′) and Vac2 (5′-CGCTCGCTTGATTGGACAGA-3′). The specificity of these primers for *H. pylori vacA* has been validated in previous studies, with reported sensitivity of 97.3% and specificity of 98.6% for clinical and environmental samples [26,35]. Cross-reactivity testing against a panel of phylogenetically related organisms (*Campylobacter jejuni*, *Campylobacter coli*, *E. coli*, *Salmonella* Typhimurium, *Pseudomonas aeruginosa*, and *Helicobacter felis*) yielded no amplification products, confirming the primers’ specificity for *H. pylori* in complex plant matrices. The analytical sensitivity of the assay was determined to be 10^2^ genome equivalents per reaction, consistent with the limits reported by Faass et al. for environmental samples. The qPCR assays were performed using the SYBR Green I fluorescent dye in a LightCycler^®^ 2.0 system (Roche Applied Science, Barcelona, Spain). The reaction mixture (20 µL) was prepared using 10 µL of qPCR Master Mix, forward and reverse primers at a final concentration of 0.8 µM, nuclease-free water, and 3 µL of template DNA. Before amplification, the DNA was normalized to a standard concentration of 10 ng/µL, following the protocol by Faass et al. [36]. The assay was conducted on a QuantStudio™ 1 Real-Time PCR System (Applied Biosystems, Thermo Fisher Scientific, USA). The thermal profile consisted of an initial activation at 95 °C for 5 min, followed by 40 cycles of denaturation at 95 °C for 30 s and annealing/extension at 58 °C for 25 s, concluding with a final extension step at 72 °C.

### 2.6. Quantitative Microbial Risk Assessment (QMRA) Framework

A static QMRA model was developed to estimate the annual probability of infection (Pann) for consumers in the Junín region, following the standard four-step framework: hazard identification, exposure assessment, dose–response modelling, and risk characterization. The exposure dose per serving (D) was estimated using a mass-balance approach:

(1) $$D=C*V*10^{-DR}$$

where C is the pathogen concentration in vegetables (CFU/g), derived from the empirical distributions obtained in this study; V is the serving size (100 g), based on regional dietary patterns; and DR represents the log-reduction associated with washing practices. In the baseline scenario, DR = 0 (no or ineffective washing). The annual exposure was estimated assuming n = 104 consumption events per year (two servings per week). To account for realistic household practices, a sensitivity analysis was conducted assuming a 1-log reduction (DR = 1), which is consistent with the reported effectiveness of plain water washing [37]. Under high contamination levels, the combination of large serving size and repeated exposure may result in high dose values (D), which can drive the dose–response models towards saturation ($P_{ind}\approx 1$). Consequently, the annual infection probability ($P_{ann}$) may approach unity for a substantial proportion of simulations, particularly for pathogens with high concentrations. This behaviour reflects a mathematical property of the model under high-exposure scenarios rather than a lack of variability in the underlying data.

Although pathogen concentrations (C) were treated as stochastic variables through Monte Carlo sampling, other exposure parameters—including serving size (V) and consumption frequency (n)—were defined as fixed values based on regional dietary patterns. This semi-probabilistic approach is commonly applied in QMRA when detailed consumption distributions are unavailable. However, it implies that variability in exposure is primarily driven by contamination levels, while inter-individual differences in consumption behaviour are not explicitly modelled.

### 2.7. Dose-Response Modeling

Specific dose-response parameters were selected from the established literature to calculate the probability of infection from a single exposure (P_ind_). For *E. coli* O157:H7 (Beta-Poisson model):

(2) Pind=1−1+DN50(21a−1)−a

Parameters: a = 0.49, N_50_ = 19.5. For *Salmonella* Typhimurium and *Shigella flexneri* (Exponential model):

(3) $$P_{ind}=1-exp(-r*D)$$

Parameters: r = 0.0052 (*S.* Typhimurium) and r = 0.0082 (*S. flexneri*). It is acknowledged that the Beta-Poisson dose-response parameters used (α = 0.49, N_50_ = 19.5) were derived from *E. coli* O157:H7 outbreak data [38], which represents a highly virulent enterohemorrhagic strain. The application of these parameters to the *E. coli* population detected in the present study (identified by culture on MacConkey agar without virulence gene screening) may overestimate infection risk if non-pathogenic or less virulent strains predominate. To address this limitation, virulence gene screening (targeting *stx1*, *stx2*, *eae*, and *hlyA*) should be incorporated in future studies to determine the proportion of pathogenic *E. coli* strains and refine dose-response parameterization accordingly [39]

### 2.8. Risk Characterization and Monte Carlo Simulation

The annual probability of infection (P_ann_) was calculated according to the independence of events principle.

(4) $$P_{ann}=1-{\left(1-P_{ind}\right)}^{n}$$

where n is the number of exposure events per year (n = 10^4^). To account for uncertainty and stochastic variability in pathogen concentrations, a Monte Carlo simulation was performed with 10,000 iterations for each pathogen. For each iteration, a concentration value (C) was randomly sampled from the experimental data. The 50th (median) and 95th percentiles of P_ann_ were calculated to characterize the risk distribution. The 5th and 95th percentiles of the simulated P_ann_ distribution were used to construct 90% confidence intervals (CI) around the median risk estimate, providing a quantitative representation of uncertainty. These uncertainty ranges were used to support the interpretation of the risk distribution. Finally, the results were benchmarked against the WHO tolerable annual risk threshold of 10^−4^. As a result, the simulated risk distributions may exhibit clustering toward extreme values (near 0 or 1), particularly under high-dose conditions, reflecting the combined effect of stochastic contamination and fixed exposure assumptions.

### 2.9. Statistical Analysis

All microbial concentration data were log-transformed (log_10_ CFU/g) prior to statistical analysis to stabilize variance and achieve normality. Descriptive statistics, including mean, range, and prevalence, were calculated for each pathogen by province and vegetable species. Non-parametric Kruskal-Wallis H-tests were performed to compare microbial concentrations across provinces. Pairwise comparisons were conducted using Dunn’s post-hoc test with Bonferroni correction. Spearman’s rank correlation coefficients (ρ) were calculated to assess co-contamination relationships between pathogens. All statistical analyses were performed using Python 3.11 (Python Software Foundation, Wilmington, DE, USA), including the libraries SciPy v1.11 (SciPy Community, USA), pandas v2.0 (pandas Development Team, USA), and statsmodels v0.14 (Statsmodels Developers, USA). Monte Carlo simulations (n = 10,000 iterations) were conducted using NumPy v1.24 (NumPy Developers, USA) with a fixed random seed (seed = 42) to ensure reproducibility.

## 3. Results

### 3.1. Overall Microbial Contamination Patterns

Biochemical analyses revealed the presumptive presence of *H. pylori* and *C. jejuni* in fresh vegetables. Specifically, *H. pylori* was identified in samples of *L. sativa* (Jauja) and *A. schoenoprasum* (Huancayo) through positive urease, oxidase, and catalase tests. Meanwhile, *C. jejuni* was detected in *L. sativa* (Chupaca and Huancayo) and *D. carota* (Huancayo), showing positivity for oxidase, catalase, nitrate reduction, and hippurate. Fecal indicator bacteria (*E. coli*) were detected in 72 of the 86 samples (83.7%), with the mean contamination levels ranging from 172.3 CFU/g in Huancayo to 217.2 CFU/g in Concepción (Table 1).

Huancayo exhibited the highest prevalence of *E. coli* (95.45%, 21/22 samples), while Chupaca showed the lowest (68.18%, 15/22 samples). In contrast, *H. pylori* and *C. jejuni* showed marginal prevalences of 2.27% and 3.41%, respectively, with detections restricted to leafy and root vegetables at specific points of sale. Specifically, the presumptive presence of *H. pylori* was identified in *L. sativa* and *A. schoenoprasum*, while *C. jejuni* was found in samples of *L. sativa* and *D. carota*. Although biochemical tests for urease (19.3%) and catalase (42%) supported these findings, molecular characterization by qPCR for *H. pylori* was negative (Table S2).

The discrepancy between the biochemical presumptive positivity and negative qPCR results may be attributed to several non-exclusive mechanisms: (i) a low genomic density of *H. pylori* below the analytical sensitivity of the qPCR assay (~10^2^ genome equivalents/reaction); (ii) the viable but non-culturable (VBNC) state of the organism, in which cells retain metabolic activity (and thus enzymatic reactions such as urease) but exhibit severely degraded or inaccessible genomic DNA; (iii) the presence of PCR inhibitors in plant matrices (e.g., polyphenols, polysaccharides) that may suppress amplification despite successful DNA extraction; and (iv) the possibility that urease-positive biochemical results reflect the activity of other urease-producing bacteria (e.g., *Proteus mirabilis*, *Klebsiella pneumoniae*) that were not excluded by the selective culture medium.

**Figure 3:**
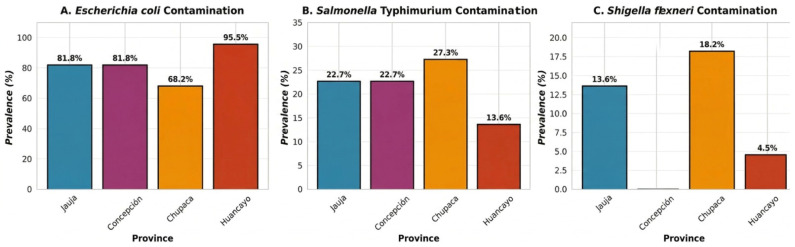
Pathogen prevalence by province. (**A**) *Escherichia coli* contamination, (**B**) *Salmonella* Typhimurium contamination, and (**C**) *Shigella flexneri* contamination across Jauja, Concepción, Chupaca, and Huancayo.

The distribution of *E. coli* contamination was highly heterogeneous, with concentrations spanning from 0 to 2700 CFU/g and a marked skew toward leafy vegetables and those typically consumed raw (Figure 3A). *Salmonella* Typhimurium was detected in 19 of 86 samples (22.1%), with prevalence varying across provinces: Chupaca (27.27%), Jauja (22.73%), Concepción (22.73%), and Huancayo (13.64%) (Table 1, Figure 3B). Mean contamination levels for *S.* Typhimurium were substantially lower than those of *E. coli*, ranging from 8.6 to 26.8 CFU/g. Notably, *S.* Typhimurium contamination was concentrated in specific high-risk vegetables, particularly *S. oleracea* (spinach) and *D. carota*, which together accounted for 45.5% of all positive detections. *S. flexneri* exhibited the lowest overall prevalence (8/86; 9.3%) and was entirely absent from the Concepción province (Table 1, Figure 3C). Chupaca showed the highest prevalence (18.18%), followed by Jauja (13.64%) and Huancayo (4.55%). The mean contamination levels ranged from 0.0 to 12.3 CFU/g. *S. flexneri* contamination was almost exclusively associated with leafy vegetables, particularly lettuce (*L. sativa*) and spinach (*S. oleracea*), suggesting specific ecological or handling-related risk factors in these crops.

### 3.2. Vegetable-Specific Contamination Profiles

**Figure 4:**
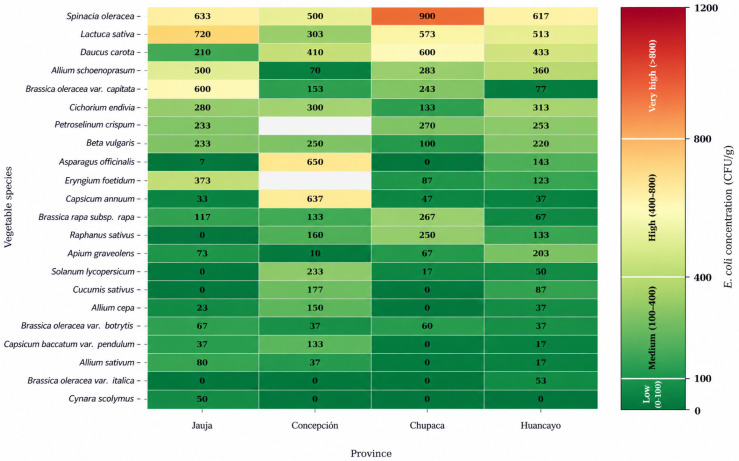
Heatmap of *Escherichia coli* contamination by province and vegetable species. Color intensity represents the mean *E. coli* concentration (CFU/g), with a quantifiable scale ranging from 0 (white) to ≥1200 CFU/g (dark red) as indicated by the color bar.

The analysis of microbial loads across different vegetable species revealed substantial variability in contamination levels (Table 2, Figure 4). *Spinacia oleracea* exhibited the highest mean *E. coli* concentration (662.5 CFU/g), followed by *Lactuca sativa* (527.5 CFU/g) and *Daucus carota* (413.3 CFU/g). These species also showed comparatively elevated *S.* Typhimurium levels, particularly *S. oleracea* (33.3 CFU/g) and *D. carota* (19.2 CFU/g).

Leafy vegetables consistently presented higher microbial burdens across all three pathogen groups. Species such as *A. schoenoprasum* and *B. oleracea* var. capitata showed intermediate *E. coli* loads (303.3 and 268.3 CFU/g, respectively), although with a lower prevalence of *S. Typhimurium* and *S. flexneri*. Conversely, lower contamination levels were observed in vegetables with protective outer layers or those with growth habits further from the ground, such as *C. scolymus* and *B. oleracea* var. *italica*.

### 3.3. Biochemical Characterization and Pathogen Identification

**Figure 5:**
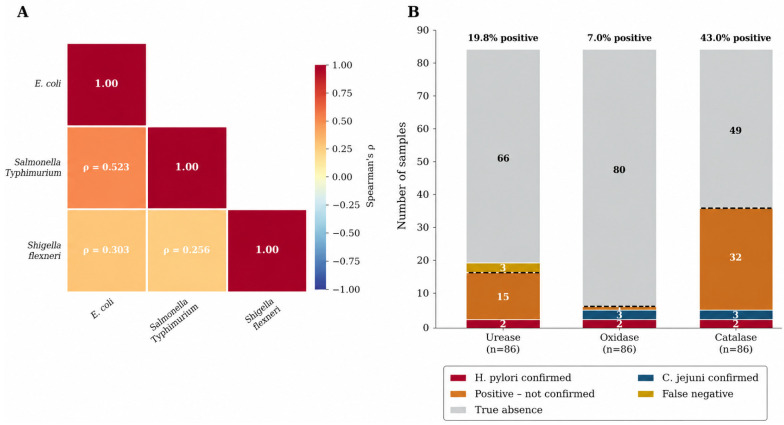
Pathogen correlation matrix and biochemical distribution. (**A**) Spearman correlation heatmap between *Escherichia coli*, *Salmonella* Typhimurium and *Shigella flexneri* (**B**) Stacked bar chart showing the distribution of positive and negative biochemical test results for urease, oxidase, and catalase across the sample set (N = 86), with bars stratified by outcome: confirmed *H. pylori*, confirmed *C. jejuni*, positive but unconfirmed (presumptive), and negative. This stratification enables visual differentiation between true pathogen-associated positivity and non-specific biochemical reactions.

Biochemical characterization was conducted on all isolates to evaluate their metabolic profiles and identify specific enteric pathogens (Figure 5B). Urease activity was observed in 19.76% (n = 17) of the 86 samples, while oxidase and catalase activities were detected in 6.98% (n = 6) and 43.02% (n = 37), respectively. For oxidase-positive isolates, supplementary tests for nitrate reduction and hippurate hydrolysis were performed, with 50% of these samples yielding positive results for both tests. Based on concordant biochemical profiles, pathogen identification was achieved in 5.81% (n = 5) of the total samples (Table 3).

Phenotypic characterization through biochemical profiling confirmed the presumptive presence of high-priority public health pathogens, highlighting the isolation of *H. pylori* and *C. jejuni* in fresh vegetables intended for raw consumption (Table S2). Quantitative analysis revealed that *E. coli* exhibited the highest microbial load across all provinces, reaching a maximum mean density of 720.00 ± 207.80 CFU/g in *L. sativa* samples from Jauja, suggesting persistent fecal contamination throughout the supply chain. Conversely, the detection of *S.* Typhimurium and *S. flexneri* despite lower mean counts (maxima of 56.67 and 33.33 CFU/g, respectively), represents a critical risk due to their low infectious doses. The high standard deviation observed, particularly in *L. sativa* from Chupaca (SD = 440.60), evidences a heterogeneous distribution of contamination, likely linked to irregular irrigation practices or deficient post-harvest handling. These findings underscore the microbiological vulnerability of fresh produce in the central region of Peru and the urgent need to implement surveillance systems based on quantitative risk assessment.

### 3.4. Pathogen Co-Occurrence and Correlation Analysis

Spearman correlation analysis revealed significant positive associations among the three pathogen groups (Figure 5A). *E. coli* and *S.* Typhimurium exhibited a moderate-to-strong correlation (rho = 0.524, *p* < 0.001), suggesting shared contamination pathways or common environmental sources. In contrast, *E. coli* and *S. flexneri* showed a weaker but significant association (rho = 0.304, *p* = 0.004), while the correlation between *S.* Typhimurium and *S. flexneri* was the least pronounced (rho = 0.258, *p* = 0.015). These correlation patterns indicate that fecal contamination events in the region likely introduce multiple pathogen types simultaneously, potentially via untreated irrigation water or organic fertilization (manure).

### 3.5. Quantitative Microbial Risk Assessment (QMRA)

**Figure 6:**
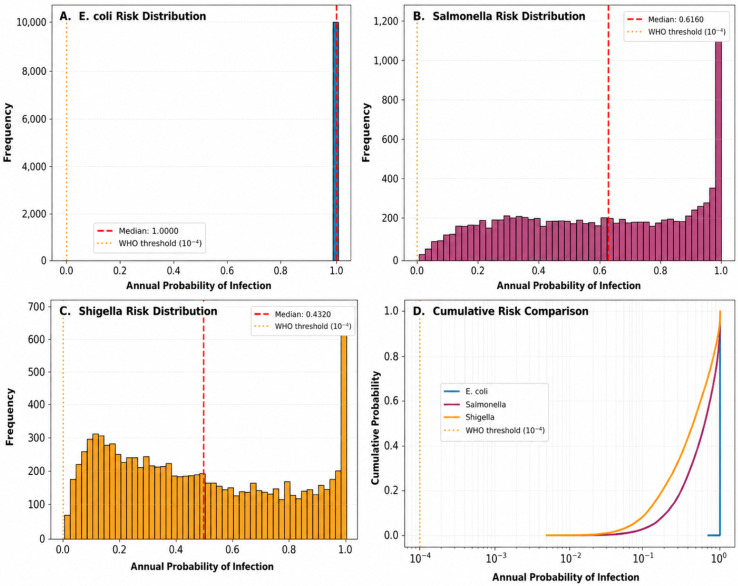
Monte Carlo-based QMRA results show the distribution of annual infection probability (Pann) for enteric pathogens. (**A**–**C**) Histograms of Pann for *Escherichia coli*, *Salmonella Typhimurium*, and *Shigella flexneri*. Red dashed lines indicate median values; orange dotted lines represent the WHO tolerable risk threshold (10^−4^). (**D**) Cumulative distribution functions showing the proportion of simulations exceeding the WHO threshold (84.3%, 22.4%, and 9.1%, respectively).

A Monte Carlo simulation (n = 10,000 iterations) was employed to estimate the annual probability of infection (P_ann_) resulting from the consumption of contaminated vegetables. The exposure scenario assumed a serving size of 100 g and a consumption frequency of two servings per week (10^4^ exposures per year). Dose–response models were parameterized using the established literature’s values: a Beta-Poisson model for *E. coli* O157:H7 (α = 0.49, N_50_ = 19.5) and exponential models for *S.* Typhimurium (r = 0.0052) and *S. flexneri* (r = 0.0082) (Table 4; Figure 6A–C).

For *E. coli*, the annual infection probability showed a highly skewed distribution, with a median value of 1.000 (IQR: 1.000–1.000) and a mean of 0.843. A total of 84.3% of Monte Carlo iterations exceeded the WHO tolerable risk threshold (10^−4^), indicating a critical level of public health concern. The 95th percentile reached 1.000, reflecting a near-certainty of infection under high-exposure scenarios. In contrast, *Salmonella* Typhimurium exhibited a markedly different distribution. Although the median Pann was 0.000, the mean value reached 0.225, reflecting a right-skewed distribution with occasional high-risk outcomes. Only 22.4% of simulations exceeded the WHO threshold, indicating that the risk is intermittent but significant under certain exposure conditions.

Similarly, *Shigella flexneri* presented a median Pann of 0.000 and a mean of 0.091, with 9.1% of iterations exceeding the WHO benchmark. This indicates a lower but non-negligible risk, driven by sporadic high-dose exposure events. The cumulative distribution functions (Figure 6D) further illustrate these differences. While *E. coli* rapidly approaches unity across simulations, reflecting consistently high exposure levels, *S. Typhimurium* and *S. flexneri* show more gradual increases, indicating heterogeneous and probabilistic risk patterns. Overall, although all pathogens exceeded the WHO safety threshold in a proportion of simulations, the magnitude and frequency of exceedance varied substantially among pathogens.

## 4. Discussion

### 4.1. High Prevalence of Enteric Pathogens in High-Altitude Andean Vegetables

The present study documented alarmingly high levels of fecal indicator bacteria and enteric pathogens in fresh vegetables from four provinces in the Junín region of Peru, situated at elevations between 3200 and 3400 m above sea level. The overall *E. coli* prevalence of 83.7% substantially exceeds rates reported in similar studies from lower-altitude regions, including 80% in Accra, Ghana (Antwi-Agyei et al., 2015), 65% in Mymensingh, Bangladesh [40], and 45–60% in various European studies [41].

This elevated contamination burden likely reflects a confluence of factors specific to high-altitude Andean agriculture. Principal among these is the reliance on untreated irrigation water sourced from glacial melt and mountain streams, which are often subject to upstream fecal contamination. Additionally, the intensive use of animal manure as fertilizer (necessitated by limited access to synthetic alternatives) and socioeconomic constraints that impede the adoption of good agricultural practices further exacerbate the microbial load on crops. The identification of *H. pylori* in vegetable samples (*L. sativa* and *A. schoenoprasum*) from Jauja and Huancayo is particularly significant. To our knowledge, this represents the first documented detection of *H. pylori* on fresh vegetables within a high-altitude Andean context.

It is important to acknowledge that the hypothesis of altitude-driven *H. pylori* survival in vegetable matrices remains speculative and is not directly supported by experimental evidence from the present study. Chen et al. [42] proposed that altitude-related hypoxia and low temperatures might enhance the survival of *H. pylori* in water and soil matrices; however, this hypothesis has not been validated in controlled field experiments. Future studies should include controlled survival experiments in Andean soil and irrigation water matrices at different altitudes, combined with environmental sampling of irrigation sources and market wash water, to establish the environmental reservoir role of *H. pylori* in high-altitude agricultural systems. Such data would be essential for refining QMRA models and informing targeted interventions.

In Andean populations, the prevalence of *H. pylori* infection is notably high, ranging from 70% to 90% [43]. However, the environmental pathways for this transmission remain poorly understood. Chen et al. [42] hypothesized that altitude-related hypoxia and low temperatures might enhance the survival of *H. pylori* in water and soil matrices. Our findings provide preliminary evidence supporting a foodborne transmission route via contaminated vegetables.

Furthermore, the detection of *C. jejuni* in lettuce and carrot samples across Chupaca and Huancayo aligns with global trends of produce contamination [26,44,45]. In contrast to the contamination levels documented in the present study, European regulatory frameworks have achieved substantially lower microbial burdens in fresh produce through the systematic implementation of GMP, GHP, and HACCP. For instance, Abadias et al. [41] reported *E. coli* prevalences of 45–60% in Spanish retail produce, with most positive samples below the 100 CFU/g threshold. The EU’s farm-to-fork approach, which includes mandatory pre-harvest water quality testing (maximum 1000 CFU/100 mL *E. coli* in irrigation water; EC Regulation 2073/2005), controlled manure application intervals (minimum 60 days before harvest for raw-consumed crops), and cold-chain traceability requirements, represents a comprehensive model that has been associated with a 40–60% reduction in produce-associated foodborne illness over the past two decades [46]. Adapting elements of this framework—particularly irrigation water quality standards and composting requirements—to the socioeconomic and infrastructural realities of the Junín highlands represents a priority for future policy development.

### 4.2. Inefficacy of Current Household Washing Practices

The QMRA results showed that risk exceedance was pathogen dependent. *E. coli* presented the highest concern, with 84.3% of simulations exceeding the WHO threshold, whereas *S. Typhimurium* and *S. flexneri* exceeded the threshold in 22.4% and 9.1% of simulations, respectively. This indicates a critical but heterogeneous risk profile rather than uniform exceedance across all pathogens. Experimental studies have demonstrated that household washing can achieve 1–4 log reductions in bacterial loads, depending on the washing agent, contact time, and produce characteristics [46]. In the present study, mean *E. coli* contamination levels ranged from 172.3 to 217.2 CFU/g, with maximum levels reaching 1200 CFU/g. Even assuming a 2-log reduction from household washing with chlorinated water, residual contamination would remain at approximately 1.7–2.2 CFU/g, which is still sufficient to pose a substantial infection risk given the low infectious dose of *E. coli* O157:H7 (N_50_ = 19.5 organisms) [47].

### 4.3. Molecular Detection Versus Biochemical Methods: Sensitivity and Specificity Considerations

The present study relied on traditional biochemical testing (urease, oxidase, catalase, nitrate reduction, hippurate hydrolysis) to identify *H. pylori* and *C. jejuni*, achieving definitive identification in only 5 of 86 samples (5.81%). This low identification rate does not necessarily reflect low pathogen prevalence but rather the inherent limitations of biochemical methods. Molecular methods, particularly PCR targeting species-specific genes (*ureA* and *glmM* for *H. pylori*; *hipO* and *glyA* for *C. jejuni*), offer superior sensitivity (95–100%) and specificity (98–100%) compared to biochemical methods [48]. The superiority of molecular techniques over biochemical methods for pathogen detection in food matrices is well established [26,35,49,50]. For future surveillance studies in the Junín region, we recommend the implementation of multiplex qPCR panels targeting: (i) virulence genes of *H. pylori* (*cagA*, *vacA*, *oipA*) to assess oncogenic potential; (ii) virulence determinants of *C. jejuni* (*cdtA*, *cdtB*, *cdtC*, *flaA*) to characterize pathogenicity; and (iii) pathotype-specific genes of *E. coli* (*stx1*, *stx2*, *eae*, *hlyA*, *elt*, *est*) to differentiate EHEC, ETEC, and EPEC strains. Such data would enable strain-specific dose-response parameterization and substantially improve the accuracy of QMRA risk estimates.

### 4.4. Public Health Implications and Food Safety

The WHO/FAO microbiological risk assessment framework establishes a tolerable annual infection risk of 10^−4^ (1 in 10,000) as a benchmark for food safety interventions [51,52]. The present study found that 84.3% of Monte Carlo simulations exceeded the 10^−4^ threshold for *E. coli*, and 22.4% and 9.1% for *S.* Typhimurium and *S. flexneri*, respectively. These findings indicate that the current vegetable supply chain in Junín poses an unacceptable public health risk, which is consistent with QMRA studies from other low- and middle-income countries [53,54].

### 4.5. Irrigation Water and Manure as Primary Contamination Pathways

The strong correlations among *E. coli*, *Salmonella*, and *Shigella* (ρ = 0.258–0.524) suggest shared contamination sources, most likely irrigation water and animal manure. It is acknowledged that the present study did not include direct microbiological analysis of water irrigation and manure samples from the study sites. This represents a limitation in establishing a definitive causal link between contamination sources and vegetable contamination. The authors relied on published data from analogous Andean contexts [55,56] and the strong inter-pathogen correlations observed in vegetable samples as indirect evidence of shared fecal contamination pathways. Future studies should incorporate paired samplings of irrigation water (at point of application) and manure (at point of use) to enable source attribution modelling. Furthermore, the traceability of vegetable origin from farm to market point of sale was not feasible within the scope of the present study, as vegetables in traditional Peruvian markets are frequently sourced from multiple farms across different micro-watersheds. A supply chain traceability study, potentially using isotopic or genetic fingerprinting of microbial isolates, is recommended to fully characterize contamination pathways in the Junín region.

Interventions targeting irrigation water quality and manure management could substantially reduce contamination. Hamilton et al. [57] demonstrated that switching from untreated wastewater to treated effluent (with ≤10^3^ *E. coli*/100 mL) reduced vegetable contamination by 2–3 logs. Drip irrigation, which minimizes direct contact between water and edible plant parts, has been shown to reduce *E. coli* contamination by 1–2 logs compared to overhead sprinkler systems [58]. Composting manure at >55 °C for ≥15 days achieves > 5-log reductions in *E. coli* and *S. Typhimurium* [59].

## 5. Conclusions

The QMRA showed a heterogeneous risk profile, with 84.3%, 22.4%, and 9.1% of simulations exceeding the WHO tolerable annual infection risk threshold for *E. coli*, *S.* Typhimurium, and *S. flexneri*, respectively. The identification of *H. pylori* and *C. jejuni* in raw-consumed vegetables underscores the potential for foodborne transmission of these serious pathogens in Andean communities. The current household washing practices are demonstrably inadequate to mitigate risk, necessitating urgent implementation of pre-harvest and post-harvest interventions targeting irrigation water, manure management, and market hygiene.

To address the identified contamination burden, the following evidence-based good production practices are specifically recommended for implementation in the Junín highlands: (1) irrigation water treatment— adoption of simple, low-cost treatment technologies such as solar disinfection (SODIS), slow sand filtration, or UV disinfection at the point of application, targeting an E. coli concentration of ≤1000 CFU/100 mL in irrigation water consistent with the WHO Guidelines; (2) manure management—mandatory thermophilic composting (>55 °C for ≥15 days) prior to field application, with a minimum 60-day pre-harvest application interval for raw-consumed crops; (3) market hygiene—implementation of potable water access points and single-use washing basins at market stalls, combined with regular E. coli testing of market wash water; (4) traceability—development of a farm-to-market traceability system using simple paper-based or digital records to enable rapid source attribution in the event of a foodborne outbreak; and (5) training—capacity-building program for smallholder farmers and market vendors on GHP and HACCP principles, adapted to the Andean context. These measures are technically feasible, cost-effective, and directly responsive to the contamination pathways identified in the present study.

The present findings have implications beyond the Junín region. The collaboration of government bodies (MINSA, SENASA), international agencies (WHO, FAO, PAHO), and academic researchers is essential for translating these results into actionable policy. Specifically, the data support the case for: (i) updating Peru’s national microbiological criteria for fresh produce (currently established under MINSA Resolution RM 591-2008/MINSA) to include criteria for *Salmonella* and *Campylobacter*; (ii) establishing a national QMRA-based fresh produce surveillance program; and (iii) integrating food safety education into agricultural extension services for Andean smallholder farmers. International collaboration with institutions conducting analogous research in high-altitude contexts (e.g., Bolivia, Ecuador, Ethiopia) would facilitate the development of region-specific risk management frameworks.

Future research should prioritize molecular characterization of pathogen strains, source attribution studies, and longitudinal surveillance to inform evidence-based policy. The public health burden of foodborne illness in Junín and more broadly, in high-altitude regions of the developing world, remains poorly quantified but is likely substantial. Addressing this burden requires coordinated action among governments, international agencies, researchers, and farming communities.

## Figures and Tables

**Table 1:** Prevalence and mean concentration of *E. coli*, *Salmonella* Typhimurium, and *Shigella flexneri* in fresh vegetables by province, Central Highlands of Peru.

Province	n	*E. coli* Positive (%)	Mean *E. coli* (CFU/g) ± SD	*Salmonella* Typhimurium Positive (%)	Mean *Salmonella* (CFU/g) ± SD ^b^	*Shigella flexneri* Positive (%)	Mean *Shigella* (CFU/g) ± SD ^b^
Jauja	22	18 (81.8%)	194.1 ± 229.6 ^a^	5 (22.7%)	30.0 ± 17.5 ^a^	3 (13.6%)	16.7 ± 5.8 ^a^
Concepción	20	18 (90.0%)	217.2 ± 198.4 ^a^	5 (25.0%)	12.7 ± 9.2 ^a^	0 (0.0%)	—
Chupaca	22	15 (68.2%)	177.1 ± 239.1 ^a^	6 (27.3%)	32.8 ± 16.9 ^a^	4 (18.2%)	22.5 ± 1.7 ^a^
Huancayo	22	21 (95.5%)	172.3 ± 174.8 ^a^	3 (13.6%)	23.3 ± 11.5 ^a^	1 (4.5%)	33.3 ^a^
Total	86	72 (83.7%)	189.5 ± 208.8	19 (22.1%)	25.5 ± 16.2	8 (9.3%)	21.7 ± 7.5
Kruskal-Wallis		H = 1.98	*p* = 0.577	H = 1.51	*p* = 0.681	H = 5.03	*p* = 0.170

Values are mean ± standard deviation. Means followed by the same lowercase letter within each column are not significantly different (Kruskal-Wallis H-test; Dunn’s post-hoc test with Bonferroni correction; α = 0.05). ^a^ All pairwise comparisons are non-significant (*p* > 0.05 after Bonferroni correction). ^b^ Mean calculated from positive samples only. CFU: colony-forming units and SD: standard deviation.

**Table 2:** Total mean and maximum concentration of enteric pathogens (CFU/g) by vegetable species.

Vegetable Species	n	Mean *E. coli* (CFU/g) ± SD	Max *E. coli* (CFU/g)	Mean *Salmonella* (CFU/g) ^a^	Mean *Shigella* (CFU/g) ^a^	Risk Category ^b^
*Spinacia oleracea*	4	662.5 ± 169.1	900	33.3	10.8	High
*Lactuca sativa*	4	527.5 ± 172.8	720	14.2	18.3	High
*Daucus carota*	4	413.3 ± 159.8	600	19.2	0	High
*Allium schoenoprasum*	4	303.3 ± 179.6	500	4.2	0	Moderate-High
*Brassica oleracea* var. *capitata*	4	268.3 ± 231.4	600	2.5	0	Moderate-High
*Cichorium endivia*	4	256.7 ± 83.4	313	0	0	Moderate-High
*Petroselinum crispum*	3	252.2 ± 18.4	270	0	0	Moderate-High
*Beta vulgaris*	4	200.8 ± 68.3	250	11.7	2.5	Moderate
*Asparagus officinalis*	4	200.0 ± 307.2	650	1.7	0	Moderate
*Eryngium foetidum*	3	194.4 ± 156.0	373	26.7	0	Moderate
*Capsicum annuum*	4	188.3 ± 298.9	637	2.5	0	Moderate
*Brassica rapa* subsp. *rapa*	4	145.8 ± 85.4	267	0	5.8	Moderate
*Raphanus sativus*	4	135.8 ± 103.4	250	9.2	0	Moderate
*Apium graveolens*	4	88.3 ± 81.8	203	0	0	Low
*Solanum lycopersicum*	4	75.0 ± 107.6	233	0	0	Low
*Cucumis sativus*	4	65.8 ± 84.4	177	1.7	0	Low
*Allium cepa*	4	52.5 ± 66.7	150	0	0	Low
*Brassica oleracea* var. *botrytis*	4	50.0 ± 15.6	67	0	0	Low
*Capsicum baccatum* var. *pendulum*	4	46.7 ± 59.7	133	0	5.8	Low
*Allium sativum*	4	33.3 ± 34.5	80	0	0	Low
*Brassica oleracea* var. *italica*	4	13.3 ± 26.7	53	0	0	Low
*Cynara scolymus*	4	12.5 ± 25.0	50	0	0	Low

Note: ^a^ Values represent mean concentrations (CFU/g) calculated from positive samples only; zero values indicate non-detection. ^b^ Risk categories were defined based on *E. coli* contamination levels according to established microbiological criteria.

**Table 3:** Biochemical identification of *Helicobacter pylori* and *Campylobacter jejuni* species and quantitative distribution (CFU/g) of enteric pathogens in fresh vegetables by sampling location.

Province	Scientific Name	Pathogen Identified	Qualitative Analysis					Quantitative Analysis (CFU/g)		
			Urease	Oxidase	Catalase	Nitrate Reduction	Hippurate	*E. coli* Mean ± SD	*Salmonella* Mean ± SD	*Shigella* Mean ± SD
Jauja	*Lactuca sativa*	*H. pylori*	+	+	+			720.00 ± 207.80	0.00 ± 0.00	20.00 ± 26.46
Chupaca	*Lactuca sativa*	*C. jejuni*	-	+	+	+	+	573.30 ± 440.60	56.67 ± 11.55	20.00 ± 26.46
Huancayo	*Daucus carota*	*C. jejuni*	-	+	+	+	+	433.30 ± 288.70	16.67 ± 28.87	0.00 ± 0.00
Huancayo	*Lactuca sativa*	*C. jejuni*	-	+	+	+	+	513.30 ± 388.60	0.00 ± 0.00	33.33 ± 57.74
Huancayo	*Allium schoenoprasum*	*H. pylori*	+	+	+			360.00 ± 79.40	0.00 ± 0.00	0.00 ± 0.00

Note: “+” indicates a positive result (presence of the tested biochemical reaction or pathogen), while “–” indicates a negative result (absence).

**Table 4:** Quantitative microbial risk assessment of annual infection probability (P_ann_) for enteric pathogens compared with the WHO tolerable risk threshold (10^−4^), including 90% uncertainty intervals (5th–95th percentiles).

Pathogen	Dose-Response Model	Model Parameters	Serving Size (g)	Median P_ann_	Mean P_ann_	90% CI (P5–P95)	% Iterations Exceeding WHO Threshold (10^−4^)	Risk Classification
*Escherichia coli* O157:H7	Beta-Poisson	α = 0.49; N_50_ = 19.5	100	1	0.843	[0.000–1.000]	84.30%	Critical
*Salmonella* Typhimurium	Exponential	r = 0.0052	100	0	0.225	[0.000–1.000]	22.40%	High
*Shigella flexneri*	Exponential	r = 0.0082	100	0	0.091	[0.000–1.000]	9.10%	Moderate

Note: The *E. coli* risk estimate was modelled using O157:H7 dose–response parameters as a conservative scenario; virulence genes were not screened.

## Data Availability

The original contributions presented in this study are included in the article/Supplementary Material. Further inquiries can be directed to the corresponding author.

## Supplementary Materials

The following supporting information can be downloaded at: https://www.mdpi.com/article/10.3390/foods15091596/s1, Table S1. Taxonomic and morphological classification of vegetables collected in the study area based on the edible part. Table S2. Biochemical profiling and phenotypic characterization of bacterial isolates in commercialized fresh vegetables.
