# Supplementary material for: Quantitative Microbial Risk Assessment of Helicobacter pylori and Enteric Pathogens in Fresh Vegetables in the Central Highlands of Peru
Source: Foods. 2026 May 5;15(9):1596. doi: 10.3390/foods15091596 (PMC13163335; doi:10.3390/foods15091596)
Supplement: Supplementary file 1 [file foods-15-01596-s001.zip › foods-4258536-supplementary.pdf]

**Supplementary material**

**Quantitative microbial risk assessment of *Helicobacter pylori* and enteric pathogens in fresh vegetables in the Central Highlands of Peru**

**Table S1.** Taxonomic and morphological classification of vegetables collected in the study area based on the edible part.

| Family | Species (Scientific Name) | Common Name | Edible Part |
|---|---|---|---|
| **Amaranthaceae** | *Beta vulgaris* | Beet | Root |
| | *Spinacia oleracea* | Spinach | Leaves |
| **Amaryllidaceae** | *Allium cepa* | Onion | Bulb |
| | *Allium sativum* | Garlic | Bulb |
| | *Allium schoenoprasum* | Scallion | Bulb |
| **Apiaceae** | *Apium graveolens* | Celery | Stem |
| | *Daucus carota* | Carrot | Root |
| | *Eryngium foetidum* | Cilantro | Leaves |
| | *Petroselinum crispum* | Parsley | Leaves |
| **Asparagaceae** | *Asparagus officinalis* | Asparagus | Stem |
| **Asteraceae** | *Cichorium endivia* | Romaine lettuce | Leaves |
| | *Cynara scolymus* | Artichoke | Inflorescence |
| | *Lactuca sativa* | Lettuce | Leaves |
| **Brassicaceae** | *Brassica oleracea* var. *botrytis* | Cauliflower | Inflorescence |
| | *Brassica oleracea* var. *capitata* | Cabbage | Leaves |
| | *Brassica oleracea* var. *italica* | Broccoli | Inflorescence |
| | *Brassica rapa* subsp. *rapa* | Turnip | Root |
| | *Raphanus sativus* | Radish | Root |
| **Cucurbitaceae** | *Cucumis sativus* | Cucumber | Fruit |
| **Solanaceae** | *Capsicum annuum* | Paprika | Fruit |
| | *Capsicum baccatum* var. *pendulum* | Yellow chili pepper | Fruit |
| | *Solanum lycopersicum* | Tomato | Fruit |

**Table S2.** Biochemical profiling and phenotypic characterization of bacterial isolates in commercialized fresh vegetables.

| Common Name | Scientific Name | Code | Urease | Oxidase | Catalase | Nitrate reduction | Hippurate | Pathogen |
|---|---|---|---|---|---|---|---|---|
| Jauja | *Cichorium endivia* | 1A | - | - | - | | | |
| | *Brassica oleracea var. botrytis* | 2A | - | - | - | | | |
| | *Brassica oleracea var. capitata* | 3A | - | - | - | | | |
| | *Cynara scolymus* | 4A | - | - | - | | | |
| | *Brassica oleracea var. italica* | 5A | - | - | - | | | |
| | *Raphanus sativus* | 6A | - | - | - | | | |
| | *Daucus carota* | 7A | + | - | - | | | |
| | *Spinacia oleracea* | 8A | - | - | - | | | |
| | *Cucumis sativus* | 9A | - | - | - | | | |
| | *Capsicum annuum* | 11A | - | - | - | | | |
| | *Lactuca sativa* | 12A | + | + | + | | | *H. pylori* |
| | *Allium schoenoprasum* | 13A | - | - | - | | | |
| | *Allium cepa* | 14A | - | - | - | | | |
| | *Allium sativum* | 15A | - | - | - | | | |
| | *Asparagus officinalis* | 16A | -- | - | - | | | |
| | *Apium graveolens* | 17A | - | - | - | | | |
| | *Capsicum baccatum var. pendulum* | 19A | + | - | - | | | |
| | *Solanum lycopersicum* | 20A | + | - | - | | | |
| | *Brassica rapa subsp. rapa* | 21A | - | - | - | | | |
| | *Beta vulgaris* | 22A | - | - | - | | | |
| | *Eryngium foetidum* | 23A | + | - | - | | | |
| | *Petroselinum crispum* | 24A | - | - | - | | | |
| Concepción | *Cichorium endivia* | 1B | - | - | + | | | |
| | *Brassica oleracea var. botrytis* | 2B | - | - | - | | | |
| | *Brassica oleracea var. capitata* | 3B | - | - | + | | | |
| | *Cynara scolymus* | 4B | - | - | - | | | |

| | | | | | | | | |
|---|---|---|---|---|---|---|---|---|
| | *Brassica oleracea var. italica* | 5B | - | - | - | | | |
| | *Raphanus sativus* | 6B | - | - | - | | | |
| | *Daucus carota* | 7B | - | - | - | - | - | |
| | *Spinacia oleracea* | 8B | - | - | - | | | |
| | *Cucumis sativus* | 9B | + | - | - | | | |
| | *Capsicum annuum* | 11B | - | - | - | | | |
| | *Lactuca sativa* | 12B | - | - | + | | | |
| | *Allium schoenoprasum* | 13B | + | - | - | | | |
| | *Allium cepa* | 14B | - | - | - | | | |
| | *Allium sativum* | 15B | - | - | + | | | |
| | *Asparagus officinalis* | 16B | + | - | + | | | |
| | *Apium graveolens* | 17B | - | - | - | - | - | |
| | *Capsicum baccatum var. pendulum* | 19B | - | - | - | | | |
| | *Solanum lycopersicum* | 20B | + | - | - | | | |
| | *Brassica rapa subsp. rapa* | 21B | - | - | - | | | |
| | *Beta vulgaris* | 22B | - | - | - | - | - | |
| | *Eryngium foetidum* | 23B | - | - | - | | | |
| | *Petroselinum crispum* | 24B | - | - | - | | | |
| | *Cichorium endivia* | 1C | - | - | - | | | |
| | *Brassica oleracea var. botrytis* | 2C | - | - | - | | | |
| | *Brassica oleracea var. capitata* | 3C | - | - | - | | | |
| | *Cynara scolymus* | 4C | - | - | + | | | |
| | *Brassica oleracea var. italica* | 5C | - | - | - | | | |
| | *Raphanus sativus* | 6C | - | - | - | | | |
| | *Daucus carota* | 7C | + | - | + | | | |
| | *Spinacia oleracea* | 8C | - | - | + | | | |
| | *Cucumis sativus* | 9C | - | - | + | | | |
| | *Capsicum annuum* | 11C | - | - | + | | | |
| **Chupaca** | *Lactuca sativa* | 12C | - | + | + | + | + | *C. jejuni* |

| | | | | | | | | |
|---|---|---|---|---|---|---|---|---|
| | *Allium schoenoprasum* | 13C | - | + | + | | | |
| | *Allium cepa* | 14C | - | - | - | | | |
| | *Allium sativum* | 15C | - | - | + | | | |
| | *Asparagus officinalis* | 16C | -- | - | - | | | |
| | *Apium graveolens* | 17C | - | - | + | | | |
| | *Capsicum baccatum var. pendulum* | 19C | + | - | + | | | |
| | *Solanum lycopersicum* | 20C | + | - | + | | | |
| | *Brassica rapa subsp. rapa* | 21C | - | - | + | | | |
| | *Beta vulgaris* | 22C | - | - | - | | | |
| | *Eryngium foetidum* | 23C | + | - | + | | | |
| | *Petroselinum crispum* | 24C | - | - | - | | | |
| **Huancayo** | *Cichorium endivia* | 1D | - | - | + | | | |
| | *Brassica oleracea var. botrytis* | 2D | - | - | - | | | |
| | *Brassica oleracea var. capitata* | 3D | - | - | + | | | |
| | *Cynara scolymus* | 4D | - | - | + | | | |
| | *Brassica oleracea var. italica* | 5D | - | - | + | | | |
| | *Raphanus sativus* | 6D | - | - | + | | | |
| | *Daucus carota* | 7D | - | + | + | + | + | *C. jejuni* |
| | *Spinacia oleracea* | 8D | - | - | + | | | |
| | *Cucumis sativus* | 9D | + | - | + | | | |
| | *Capsicum annuum* | 11D | - | - | + | | | |
| | *Lactuca sativa* | 12D | - | + | + | + | + | *C. jejuni* |
| | *Allium schoenoprasum* | 13D | + | + | + | | | *H. pylori* |
| | *Allium cepa* | 14D | - | - | - | | | |
| | *Allium sativum* | 15D | - | - | + | | | |
| | *Asparagus officinalis* | 16D | + | - | + | | | |
| | *Apium graveolens* | 17D | - | - | - | | | |
| | *Capsicum baccatum var. pendulum* | 19D | - | - | + | | | |
| | *Solanum lycopersicum* | 20D | + | - | + | | | |

| | | | | | | | | |
|---|---|---|---|---|---|---|---|---|
| | *Brassica rapa subsp. rapa* | 21D | - | - | + | | | |
| | *Beta vulgaris* | 22D | - | - | + | | | |
| | *Eryngium foetidum* | 23D | - | - | + | | | |
| | *Petroselinum crispum* | 24D | - | - | - | | | |
